# Advances in Materials Joining and Additive Manufacturing

**DOI:** 10.3390/ma19020311

**Published:** 2026-01-13

**Authors:** Xiaochao Liu, Lei Shi

**Affiliations:** 1 School of Mechanical Engineering, Southeast University, Nanjing 211189, China; 2 School of Materials Science and Engineering, Shandong University, Jinan 250061, China; lei.shi@sdu.edu.cn

As the manufacturing sector advances toward lightweight design [1,2,3], high performance [4,5], and sustainable development [6,7,8], materials joining and additive manufacturing technologies are encountering both unprecedented opportunities and significant challenges [9,10]. Industries such as aerospace, new energy vehicles, and high-end equipment are increasingly demanding solutions for joining dissimilar materials [11,12], fabricating functionally graded structures [13,14], and ensuring long-lasting joint integrity [15,16]. These requirements are accelerating the development of key processes—including laser processing, friction stir welding, and bolted connections—toward higher precision and enhanced reliability. In this context, this Special Issue gathers cutting-edge research in materials joining and additive manufacturing, showcasing recent progress in laser-assisted processing, friction stir welding, laser cladding, and the reliability of bolted connections. The five selected contributions examine microstructure evolution, interfacial behavior, corrosion resistance, and mechanical performance across a variety of metallic systems, providing valuable insights for applications in aerospace, automotive, and structural engineering.

In the first contribution, Péter et al. [17] investigate composition profiles at metal substrate–deposit interfaces produced via laser-assisted additive manufacturing. Using energy-dispersive spectroscopy (EDS), the authors reveal an asymmetric composition transition across dissimilar metal pairs such as H13, 15-5PH, In625, and In718 coatings on stainless steel substrates. A key finding is that the composition decay follows an exponential trend, well-captured by a mathematical model based on convective mixing in the molten zone. The model suggests that the thickness of the intermixing layer—rather than full melt pool depth—governs the compositional gradient. This work provides a quantitative foundation for optimizing interfacial properties in laser-cladded components.

Kremer et al. [18] examine the influence of heat treatments on the corrosion behavior of CuSn10 tin bronze fabricated by laser powder bed fusion (LPBF). The study compares as-built samples with those treated at 320 °C, at 650 °C, and with a two-stage 800 + 400 °C process. The results indicate that, while lower-temperature treatments (320 °C and 650 °C) do not significantly alter corrosion rates, the two-stage treatment slightly improves corrosion resistance. Microstructural analysis links these changes to phase transformations and tin precipitation. The work underscores that corrosion rates remain within a consistent range (0.1–0.5 mm/year) regardless of heat treatment, supporting the use of LPBF CuSn10 in corrosive environments.

Yin et al. [19] explore the effect of ultrasonic vibration (UV) on grain microstructure in friction-stir-welded Al/Mg thin sheets. Electron backscatter diffraction (EBSD) analysis shows that UV promotes dynamic recrystallization, increasing high-angle grain boundaries and refining grains in the weld nugget zone. Although UV does not change the dominant recrystallization mechanisms—continuous dynamic recrystallization (CDRX) for Al and discontinuous/continuous DRX for Mg—it enhances interfacial bonding by reducing intermetallic compound (IMC) thickness. These microstructural improvements contribute to a 12.2% increase in ultimate tensile strength, demonstrating the potential of ultrasonic-assisted FSW for high-integrity dissimilar joints.

Wang and Jean [20] focus on optimizing the mechanical and tribological properties of laser-cladded WC/Co/Ni composites using Taguchi design and response surface methodology. The study identifies Co%, Ni%, laser power, and scanning height as key factors influencing wear volume. An interaction model successfully predicts wear behavior with high accuracy (adjusted R^2^ = 0.84). Microstructural observations reveal that coatings with optimized Co/Ni ratios exhibit reduced wear loss and improved carbide distribution. This statistical approach provides a reliable framework for designing wear-resistant coatings for demanding applications.

Yang et al. [21] address the reliability of bolted joints by proposing a methodology for predicting bolt loosening life under variable amplitude loading. Applying Miner’s linear damage rule, the authors validate their model using two-block loading tests on M6 and M8 bolts. Finite element analysis helps identify vulnerable regions in multi-bolted structures, with experimental results confirming predictions within a ±1.2× error band. This practical approach enables accurate service life estimation for bolted connections in vibrating environments, supporting maintenance and safety in mechanical assemblies.

Together, these studies underscore the importance of process control, microstructure characterization, and modeling in advancing joining and additive manufacturing technologies. From laser-cladded interfaces to ultrasonically enhanced welds and reliable bolted joints, the findings presented here offer valuable guidelines for researchers and engineers seeking to improve performance, durability, and sustainability in modern manufacturing. Looking ahead, intelligent process control and digitalization [22,23,24], multi-scale modeling and simulation [25,26], and sustainability and lifecycle engineering [27,28] remain key areas for future development.
